# Perceived stress, coping strategies, and mental health status among adolescents during the COVID-19 pandemic in Switzerland: a longitudinal study

**DOI:** 10.1007/s00787-022-02119-y

**Published:** 2022-12-14

**Authors:** Simon Foster, Natalia Estévez-Lamorte, Susanne Walitza, Shota Dzemaili, Meichun Mohler-Kuo

**Affiliations:** 1 Department of Child and Adolescent Psychiatry and Psychotherapy, Psychiatric University Hospital, University of Zurich, Zurich, Switzerland; 2 La Source, School of Nursing Sciences, HES-SO University of Applied Sciences and Arts of Western Switzerland, Lausanne, Switzerland

**Keywords:** Mental health problems, Coping strategies, Perceived stress, COVID-19 pandemic, Adolescents

## Abstract

This longitudinal, prospective study investigated associations between perceived COVID-19-related stress, coping strategies, and mental health status among adolescents during the first lockdown of the COVID-19 pandemic and one year after the lockdown in Switzerland within a large, national sample. A self-report on-line survey was completed by 553 adolescents (age-range 12–18 years in 2021) in the summers of 2020 and 2021, assessing symptoms of various mental health problems, perceived COVID-19-related stressors, and coping strategies. Overall, participants reported less COVID-19 related stress one year after the lockdown, though mental health status remained stable. ‘Challenges at home or with others’ were significantly associated with mental health problems in both genders, whereas ‘trouble getting medical care or mental health services ‘was associated with mental health problems in girls. Perceived stress and pre-existing psychiatric problem were significantly linked to all mental health outcomes at both time points. Parents’ poor relationships with partners during the lockdown was associated with increased anxiety symptoms in their children. Using cognitive restructuring to cope with stress was associated with less, while negative coping was associated with more anxiety, depression, and attention deficit hyperactivity disorder (ADHD) symptoms one year post lockdown. Girls appear to have been more affected by the pandemic than boys, with youths with pre-existing psychiatric problems especially vulnerable to its detrimental effects. Healthcare and school professionals should support to identify high-risk adolescents with negative and avoidant coping strategies and train youths to use positive coping strategies.

**Supplementary Information:**

The online version contains supplementary material available at 10.1007/s00787-022-02119-y.

## Introduction

The COVID-19 pandemic has been both an acute and chronic threat to the well-being of the general population, due to the numerous challenges posed by social disruption, including social isolation, financial insecurity, and confinement-related stress (e.g., interruptions in daily routines, important events, and plans). Adolescents might be especially vulnerable to these challenges, since they are facing significant changes in all aspects of life. In general, adolescence is considered a critical stage of life, because mental health problems that develop during this stage may have long-term negative consequences later in life [1–3]. Furthermore, compared to adults, young people are more vulnerable to their immediate environment and have fewer resources and past experiences to cope with stressful situations [4, 5]. Therefore, due to feelings of uncertainty, they may experience more difficulties overcoming the many challenges imposed by the COVID-19 pandemic, making them even more vulnerable to developing mental health problems [6]. Already, studies exploring the psychological impact of the first COVID-19 pandemic lockdown on children and adolescents have identified, among a host of other symptoms, elevated rates of depressive, anxiety, attention deficit hyperactivity disorder (ADHD), and oppositional defiant disorder (ODD) symptoms, as well as a higher prevalence of perceived psychological stress [7–14]. In this context, evidence suggests that some factors may considerably impact mental health. For instance, female gender, unfavorable family-related factors (e.g., with respect to income, quality of relationships within the family, social support), pre-existing mental health problems, and COVID-19 related stressors (e.g., fear of contagion) have all been linked to mental health problems during the lockdown period [6–8, 12, 14–18] indicating the importance of identifying risk and protective factors which may influence mental health during this unprecedented situation.

Finally, how adolescents cope with COVID-related stressors may influence their mental health outcomes. In studies that investigated the relationship between coping and mental health during the first lockdown, it seems that maladaptive strategies—such as using alcohol to reduce stress, avoiding activities to manage difficulties and disengagement coping—were related to poor mental health outcomes, whereas active coping (e.g., trying to view things in a positive light), problem-focused coping, and engagement coping (acceptance, positive thinking) were associated with fewer mental health problems and greater psychological adjustment [9, 10, 12, 13, 17, 19]. Moreover, the stress experienced by prolonged exposure to social disruptions and their related consequences may accumulate over time [20] and become more challenging and difficult for youth to manage. Under such circumstances, it is possible that more young people, including those who tended to use adaptive strategies during the first phase of the pandemic, may over time turn to maladaptive strategies to cope with stress, thereby exacerbating potential mental health problems [10].

To date, researchers have largely focused on the immediate consequences of the first lockdown on mental health outcomes and/or the few following months. Studies addressing the pandemic’s long-term effects on mental health among adolescents at later stages of the pandemic are scarce [21–23]. Additionally, to our knowledge, no studies investigated the impact of associated factors (e.g., COVID-related stressors, and coping strategies that might help to reduce or prevent the development of mental health symptoms) at later stages in Switzerland.

Therefore, the overriding aim of this study was to investigate associations between perceived COVID-19 related stress, coping strategies, and mental health status among adolescents during the first lockdown and one year after the lockdown. Like many other countries, Switzerland has been forced to implement extreme measures and enacted a total lockdown. On March 16th, 2020, schools and most shops were closed nationwide, and from March 17th onward, measures such as quarantines, social distancing or limitation of social gathering have been implemented to prevent the collapse of the health system. A gradual opening began at the end of April until a total opening on June 8th, 2020. Many schools already resumed in May 2020 with reduced hours of presence and reduced numbers of students. There was no other total lockdown in Switzerland after the first one in spring 2020, despite a large second wave with high incidences of infections in autumn and winter 2020. In-class teaching remained for primary and secondary schools throughout the whole pandemic with measures of masks wearing, social distancing and regular PCR tests in school. However, most leisure or group activities were either canceled or with restricted numbers of attendees. To this end, we first compared changes in the prevalence of COVID-related stressors, and of mental health symptoms (depression, anxiety, ADHD, ODD) reported by adolescents between the two assessments. We further investigated how perceived COVID-related stressors and their association with mental health outcomes changed over time during the pandemic. Finally, we examined the longitudinal and cross-sectional effects of COVID-related stressors and coping strategies on mental health outcomes, taking relevant factors like gender and previous mental health problems into consideration.

## Methods

### Study design

The present longitudinal study was conducted among a large national sample of adolescents 12–18 years old from all three language regions (German-, French-, and Italian-speaking) in Switzerland. The baseline survey (wave 1) was conducted from July–October 2020, its aims being to assess the impact of the first lockdown due to COVID-19 by reporting the prevalence of symptoms of mental illness and documenting the various stressful situations caused by COVID-19 perceived by adolescents. Full details of the study’s methodology have been published elsewhere [18].

The follow-up survey was conducted one year later, from July to September 2021, employing the same measures to assess changes in mental health symptoms, perceived stress, and coping strategies over time.

### Participants

For the baseline survey, we aimed to obtain a large, national community sample of 1000 adolescents 12–17 years old from all three language regions (German-, French-, and Italian-speaking) in Switzerland, collaborating with the LINK Institute and to recruit parents with children 12–17 years old through the LINK Internet Panel. The LINK Internet Panel has over 100,000 subjects representative of the internet-using Swiss population from 15 to 79 years old.

A letter of invitation that described the study was sent by email with a link to the survey platform to the parents of potentially eligible adolescents in all three language regions. At the beginning of the survey, the parents were asked (1) whether they agreed to participate in the survey; (2) whether they had children from 12 to 17 years old in their household; and (3) whether they would allow their children to participate in the survey. If parents answered “yes” to all three questions, they were asked to start the survey, which lasted approximately 15 min. Questions assessed the parents’ own mental health status and stress and the impact that the COVID-19 pandemic and lockdown had had on them. After parents completed the survey, their children could either complete the survey right away or complete it later. In total, 1146 children completed the baseline survey.

The follow-up survey was conducted one year after the first, at which time these same parents and children were re-contacted and asked to participate again and parents again indicated whether they would agree that their children participated. Six hundred forty-one adolescents from the previous sample responded to the follow-up survey (56%). However, after examining sociodemographic data on the adolescents, 88 whose age and gender were inconsistent with the prior survey were excluded from further analysis (one way such inconsistency was possible was when a household had multiple children 12–17 years old and the parent made the mistake by inviting the wrong child to participate the second time). Consequently, the final sample included 553 adolescents who had participated in both surveys.

To examine potential bias caused by non-response, we ran a multiple logistic regression predicting non-participation at follow-up. The results showed that girls were more likely to be non-participants (*p* = 0.020) and higher COVID-19-related stress was marginally predictive of non-participation (*p* = 0.054). No significant differences in any mental disorder symptom, age, language region, nationality, previous psychiatric problem, parent’s education, and parent’s depression were identified between these two groups (supplement Table 1).

**Table 1:** Stressors related to COVID-19

Prevalence of somewhat/very in %	Girls *N* = 252		Boys *N* = 301		Generalized Estimating Equations models^1^ (*P* value)		
	2020	2021	2020	2021	Time	Girl	Time*girl
1.My family has experienced financial problems	4.8	8.3	6.0	4.7	0.428	0.524	0.063
2.Unable to spend time in person with my friends or family	38.1	35.7	30.2	22.9	0.015	0.052	0.217
3.Unable to participate in social activities and normal routines	41.3	37.5	34.2	27.9	0.043	0.088	0.538
4.Having to change, postpone, or cancel important plans or events	47.2	44.8	32.6	28.9	0.243	< 0.001	0.732
5.Challenges at home or with others	9.9	15.1	5.3	6.3	0.564	0.043	0.437
6.My family has experienced trouble getting groceries or other needed supplies	5.6	3.6	3.0	1.7	0.252	0.140	0.831
7.Watching or hearing distressing news reports about COVID-19	25.0	19.4	16.0	9.0	0.004	0.009	0.242
8.Not being sure about myself or someone close to me getting COVID-19	26.2	20.2	17.9	15.0	0.241	0.020	0.653
9.Myself or someone close to me having symptoms or being diagnosed with COVID-19	26.2	21.8	20.3	13.0	0.005	0.104	0.262
10.Trouble getting medical care or mental health services	9.1	14.3	6.0	4.3	0.314	0.166	0.040
11.Not being sure about when COVID-19 will end or what will happen in the future	34.9	29.0	20.6	19.9	0.806	< 0.001	0.313
12.Difficulty completing my school/ work responsibilities online	17.1	12.4	17.7	14.6	0.225	0.852	0.597
13.Unable to complete educational or work requirements	14.7	14.7	13.6	11.3	0.317	0.721	0.485
14.Needing to take on greater family and /or work responsibilities	8.3	8.7	7.0	5.3	0.331	0.557	0.406
Sum of stress scores (means ± SD)	26.0 ± 6.5	24.9 ± 7.4	23.8 ± 6.7	22.3 ± 6.5	< 0.001	< 0.001	0.529

^1^The *p* value is derived from Generalized Estimating Equations. The Odds ratio and 95% confidence were included in the supplement Table 2

### Measures

The baseline questionnaire for the adolescents was developed in collaboration with the CORONA HEALTH APP Study, which was supported by the Robert-Koch Institute in Germany [24]. The same questionnaire, with minor revisions, was used for the follow-up survey.

To maximize the response rate, the questionnaire’s duration we kept under 20 min. To achieve this, we selected short, yet well-established screening questionnaires. The same questionnaires were used to assess the symptoms of ADHD, oppositional defiant disorder (ODD), depression and anxiety, with ADHD (4 items) and ODD (3 items) assessed using the screening questions from Kiddie Schedule for Affective Disorders and Schizophrenia (K-SADS) [25]. These screening items have been shown to be valid identifying children with ADHD or ODD [26].

Anxiety symptoms were assessed using the brief version of the Spence Children’s Anxiety Scale for Children (SCAS-C) [27]. The brief version is comparable to the full version and has good validity and reliability [27]. Its items assess children’s anxiety symptoms using a 4-point scale (never (0), sometimes (1), often (2) and always (3)). We calculated a total score by summing the eight items and created a dichotomized variable using a cut-off score of 7.5 for girls and 5.5 for boys, as recommended in previously published literature [27]. We also used three questions from the Patient Health Questionnaire (PHQ) to assess depression (PHQ-2) and sleep problems in the baseline survey. The PHQ-2 has been shown to be an effective depression screening tool. Summation scores for the two items were further dichotomized as positive versus negative with a cut-off score of 3 [28]. The longer version of the PHQ-9 [29] was administered to assess depression symptoms during the follow-up survey.

### Perceived stress

Perceived stress was measured using the first part of the Response to Stress Questionnaire (RSQ)–COVID-19 (adolescent version) that was developed by the Stress and Coping Research Lab at Vanderbilt University [30]. The first part of the questionnaire includes a checklist of 14 situations during COVID-19 that respondents sometimes find stressful or have problems dealing with. Respondents rate each specific situation in terms of how often each stress occurred during the COVID-19 lockdown period using a 4-point scale with the response options “not at all” (1), “a little” (2), “somewhat” (3), and “very” (4). We reported the percentage of respondents who reported either somewhat or very. The same list of stressful situations attributed to COVID-19 was used in the follow-up survey, when respondents were asked to rate each specific situation in terms of how often it had occurred over the preceding six months.

### Coping strategies

For coping strategies, we used the self-report version of Kidcope for adolescents (ages 13–18 years) [31]. This version includes 11 items which assess ten different coping strategies. The respondents were asked to rate their frequency of use on a 4-point (0–3), Likert-type scale (‘not at all’ to ‘almost all the time’). These strategies can be grouped into “active coping strategies” (i.e., problem solving, positive and negative emotional regulation, social support, cognitive restructuring), “avoidant coping strategies” (i.e., distraction, social withdrawal, wishful thinking, resignation), and “negative coping strategies” (i.e., self-criticism, blaming others) [32, 33]. Summation scores were calculated by adding the ratings of all items belonging to one category. We further dichotomized each item into absent (‘not at all’ or ‘seldom’) or present (‘often ‘ or ‘all the time’).

### Other related risk factors

During the baseline survey, we have asked the participants whether they had ever been diagnosed with a mental illness (“previous psychiatric problem”). Parents were additionally asked about the quality of their relationship with their partner during the lockdown. A poor relationship was defined as having a ‘bad’ or ‘very bad’ relationship with the partner.

### Data analysis

All statistical analyses were conducted using SAS version 9.4 [34]. Contingency tables were drafted to report what percentage of youth participants reported COVID-related stress, various mental health symptoms, and use of coping strategies, stratified by gender and survey year. Stratification by gender was done because previous studies investigating mental health during the pandemic reported gender differences [6, 14, 18, 35–37]. Differences over time in the prevalence of perceived stress and mental health problems from 2020 to 2021 were compared using Generalized Estimating Equations (GEE) [38, 39], which can be used to fit regression models to handle correlated outcomes when repeated measurements of the same participant have been performed over time. ‘Time’ was coded ‘0’ for baseline and ‘1’ for follow-up. The *p* value for ‘time’, derived from GEE analyses using Wald chi-square statistics [40] (Tables 1 and 2), was reported to identify whether significant change had occurred between 2020 and 2021. For perceived stress (Table 1), we have fitted a GEE model for each stressor that included time, gender and time*gender interaction effects simultaneously in the model. It would allow us to examine whether perceived COVID-related stress changed over time, varied by gender and whether there is an interaction effect such that the change of the perceived stress varied by gender. Due to the limited space of the table, we reported only *p* values in Table 1 and the odds ratios with 95% confidence intervals were included in the supplement Table 2. Final multiple regression models (Tables 5 and 6) examined the effects of perceived stress and coping strategies on the sum score of each of the mental health symptoms (pertaining to anxiety, depression, ADHD, or ODD) reported in 2021, controlling for other correlates (gender, parents’ poor relationship with partners during the lockdown, previous psychiatric problems). Note that all variables except parents’ poor relationship with partners during lockdown are based on adolescents’ reports.

**Table 2:** The prevalence of mental health outcomes

%	Girls *N* = 252			Boys *N* = 301		
	2020 (%)	2021 (%)	*P* value^3^	2020 (%)	2021 (%)	*P* value^2^
Depression, PHQ2	7.5	7.9	0.835	4.3	4.7	0.835
Depression, PHQ9^1^	N/A	15.5	N/A	N/A	5.3	N/A
Anxiety	13.9	12.3	0.450	11.6	12.6	0.662
ADHD	21.8	24.6	0.354	22.6	20.6	0.414
ODD	20.2	18.7	0.599	10.6	11.3	0.746
Any mental health disorder (in %)	38.5	38.5	0.999	33.6	29.9	0.213

Sum scores	2020 Mean ± SD	2021 Mean ± SD	*P* value^3^	2020 Mean ± SD	2021 Mean ± SD	*P* value^2^
Depression, PHQ2	0.94 ± 1.01	1.14 ± 1.20	0.008	0.65 ± 0.92	0.74 ± 0.92	0.116
Depression, PHQ9^1^	N/A	5.13 ± 4.92	N/A	N/A	3.19 ± 3.29	N/A
Anxiety	3.63 ± 3.24	3.86 ± 3.26	0.152	2.56 ± 2.44	2.55 ± 2.69	0.943
ADHD	6.83 ± 1.76	6.82 ± 1.86	0.912	6.93 ± 1.77	6.83 ± 1.72	0.297
ODD	5.10 ± 1.43	5.09 ± 1.53	0.847	4.91 ± 1.31	4.90 ± 1.35	0.860

^1^PHQ 9 was used in 2021 only

^2^The *p* value is derived from generalized estimating equations

## Results

Among the 553 adolescents who participated in both the wave 1 and wave 2 surveys, 54.4% were male and 58.0% were attending mandatory schools. Their mean age in 2021 was 15.10 years (SD: 1.67, range 12–18). About 11.8% of them had previous psychiatric problems and 4.5% of the parents reported poor relationship with their partners.

### Perceived stress & mental health status

Table 1 shows the percentages of adolescents who reported feeling “rather” or “very” stressed about various situations related to COVID-19 in 2020 and 2021. Overall, sum of perceived COVID-19-related stress scores decreased significantly in 2021 compared to 2020 for both boys and girls. Among all 14 stressors, four stressors decreased significantly from 2020 to 2021. Girls had higher sum scores of perceived COVID-related stress than boys did (see also [18]) and significantly more girls perceived stress in 5 of the 14 stressors compared to boys. Significant time and gender interaction was found in one stressor such that more girls perceived stress by ‘troubles getting medical care or mental health services’ in 2021 than in 2020 whereas less boys did so in 2021. In 2021, the three most stressful situations perceived secondary to COVID-19 by participating youths were the same as in 2020 for both girls and boys: being unable to spend time in person with friends or family, being unable to participate in social activities and normal routines, and having to change, postpone, or cancel important plans or events.

Despite the global trend of reduced stress in 2021 in girls, the percentage of perceived stress in two COVID-19 related stressors increased significantly from 2020 to 2021: challenges at home or with others increased from 9.9 to 15.1% (*p* = 0.024); and trouble getting medical care or mental health services increased from 9.1 to 14.3% (*p* = 0.028). Financial problems of the family increased from 4.8% to 8.3% as well, but this increase just fell short of statistical significance (*p* = 0.0626).

Among boys, 13 of the 14 COVID-19-related stressors in 2021 decreased as well, with four stressors which decreased statistically significant: the percentage of boys reporting stress related to being unable to spend time in person with friends or family decreased from 30.2 to 22.9% (*p* = 0.015); being unable to participate in social activities and normal routines decreased from 34.2 to 27.9% (*p* = 0.043); watching or hearing distressing news reports about COVID-19 decreased from 16.0 to 9.0% (*p* = 0.004); and concerns about “myself or someone close to me having symptoms or being diagnosed with COVID-19” decreased from 20.3 to 13.0% (*p* = 0.005). Similar to girls, perceived stress about ‘challenges at home or with others’ increased from 2020 to 2021 in boys, however, the increase is not significant.

Table 2 shows the prevalence of symptoms of depression, anxiety, ADHD, ODD, and any one of these disorders when considered together. In both 2020 and 2021, girls had a strikingly higher prevalence of ODD symptoms than boys (averaging 19.5% vs. 10.9% over the two study years) and a somewhat higher prevalence of depression symptoms (6.8% vs. 4.5%). Comparing 2020 and 2021, few changes in prevalence were evident and no change from 2020 to 2021 was statistically significant. Thus, the examined mental health problems largely remained stable from 2020 to 2021.

Against the overall trend of decreased perceived stress over time, three COVID-19-related stressors (Family financial problem, challenge at home or with others, trouble getting medical care or mental health services) increased from 2020 to 2021 among girls and, ‘challenge at home or with others’ also increased from 2020 to 2021 in boys, the question arises whether these stressors also were associated with their mental health problems and whether the association between these stressors and mental health outcomes varied by gender. We conducted two sets of logistic regression models. Model 1 (Table 3) tested the effects of the stressors and gender on each of the mental health outcomes and model 2 included additional gender*stressor interaction. All of the three stressors are independently associated with all mental health outcomes adjusting for gender. In addition, girls are more likely to have depression and ODD symptoms controlling for the stressors. No interaction were found (Table 3) partly due to the small sample size of ‘cases’ in these mental health outcomes.

**Table 3:** Association between COVID-19-related stressors in 2021 and mental health 2021

COVID-19-related stressors	Depression	Anxiety	ADHD	ODD	Any
Model 1					
Total	OR [95% CI]	OR[95% CI]	OR[95% CI]	OR[95% CI]	OR[95% CI]
My family has experienced financial problems	3.15 [1.36, 7.26]*	2.68 [1.19, 6.01]*	2.74 [1.35, 5.54]*	3.17 [1.50,6.71]*	3.04 [1.50, 6.15]*
Girl	3.12 [1.69, 5.76]*	0.92 [0.55,1.54]	1.21 [0.81, 1.81]	1.72 [1.06, 2.78]*	1.41 [0.99,2.02]
Challenges at home or with others	9.42 [4.90,18.08]*	7.98 [4.24, 15.00]*	4.68 [2.64,8.31]*	5.15 [2.82, 9.41]*	6.45 [3.45,12.06]*
Girl	2.61 [1.38, 4.96]*	0.71 [0.41, 1.23]	1.07 [0.70,1.62]	1.51 [0.92, 2.48]	1.26 [0.87,1.83]
Trouble getting medical care/mental health services	2.66 [1.27, 5.60]*	3.96 [1.98, 7.92]*	2.83 [1.52, 5.24]*	2.27 [1.14, 4.51]*	2.93 [1.60,5.39]*
Girl	2.90 [1.56, 5.39]*	0.79[0.47,1.35]	1.11 [0.74,1.68]	1.64 [1.00,2.67]*	1.32 [0.92, 1.89]
Model 2, with interaction					
My family has experienced financial problems	1.40 [0.17, 11.38]	2.98 [0.89, 10.02]	2.24 [0.72, 6.95]	4.94 [1.55, 15.75]*	4.58 [1.49, 14.07]*
Girl	2.81 [1.48, 5.35]*	0.94 [0.55,1.62]	1.17 [0.77, 1.79]	1.86 [1.12, 3.11]	1.48 [1.02, 2.15]*
Interaction	2.85 [0.28, 28.61]	0.83 [0.16,4.21]	1.40 [0.33, 5.96]	0.48 [0.11, 2.19]	0.50 [0.12,2.12]
Challenges at home or with others	12.55 [3.96,39.85]*	10.08 [3.78, 26.88]*	3.89 [1.51, 10.04]*	4.19 [1.48, 11.88]*	7.59 [2.64, 21.79]*
Girl	2.96 [1.36, 6.43]*	0.78 [0.42, 1.47]	1.02 [0.65, 1.61]	1.42 [0.82, 2.47]	1.29 [0.87, 1.90]
Interaction	0.66 [0.16, 2.66]	0.67 [0.19, 2.42]	1.34 [0.41, 4.42]	1.37 [0.38, 4.95]	0.78 [0.21, 2.87]
Trouble getting medical care/mental health services	3.56 [0.72,17.62]	3.32 [0.97, 11.37]	3.55 [1.15, 10.98]*	1.46 [0.31, 6.86]	2.88 [0.94. 8.83]
Girl	3.04 [1.56, 5.90]*	0.76 [0.43, 1.37]	1.15 [0.75, 1.78]	1.55 [0.92, 2.59]	1.31 [0.90,1.92]
Interaction	0.70 [0.12,4.22]	1.30 [0.29, 5.78]	0.72 [0.19, 2.79]	1.78 [0.31, 10.10]	1.03 [0.27, 3.90]

*P < 0.05; if the 95% CI does not contain the value 1, the *p* value is less than 0.05

As evident from the odds ratios (OR) and 95% confidence intervals (CI) shown in supplement Table 3, the stressors were consistently linked to girls’ symptoms of depression, anxiety, ADHD, and ODD. On the other hand, for boys, only ‘challenges at home or with others’ were consistently linked to mental health problems.

### Coping strategies

Table 4 shows the percentage of youths who used the various coping strategies either ‘often’ or ‘all the time’ and the mean scores for each item during the first lockdown and one year after the lockdown. As stated earlier, the 11 items were grouped into three categories: active, avoidant, negative. The coping strategies used most commonly by adolescents were ‘resignation’ and ‘cognitive restructuring’. During both assessments, girls used coping strategies like ‘wishful thinking’ and ‘emotional regulation by calming oneself down’ more frequently than boys. In 2021, boys continued to use cognitive restructuring, while girls reduced their cognitive restructuring and were more likely to cope by means of emotional regulation through the expression of feelings.

**Table 4:** Coping Strategies by gender and survey year

	Girls			Boys			Girls		Boys	
	2020	2021	*p*	2020	2021	*p*	2020	2021	2020	2021
	%	%		%	%		Mean (SE)	Mean (SE)	Mean (SE)	Mean (SE)
Active										
Cognitive restructuring	52.0	40.1	0.002	49.3	51.2	0.587	1.51 (0.83)	1.40 (0.88)	1.49 (0.92)	1.53 (0.94)
Problem solving	14.7	12.4	0.425	18.0	17.6	0.890	0.74 (0.75)	0.70 (0.73)	0.77 (0.83)	0.77 (0.80)
Emotional regulation-express feeling	6.0	7.1	0.564	3.7	2.3	0.285	0.39 (0.66)	0.39 (0.64)	0.26 (0.55)	0.24 (0.50)
Emotional regulation-calming down	16.3	18.7	0.386	10.0	11.0	0.673	0.75 (0.85)	0.76 (0.84)	0.49 (0.74)	0.57 (0.73)
Social support	23.0	25.0	0.535	19.7	19.6	0.981	0.94 (0.88)	0.94 (0.88)	0.87 (0.86)	0.86 (0.87)
Sum scores							4.33 (2.43)	4.19 (2.57)	3.88 (2.46)	3.97 (2.43)
Avoidant										
Distraction	27.0	25.8	0.696	24.0	29.6	0.069	1.07 (0.89)	1.06 (0.83)	0.94 (0.86)	1.00 (0.88)
Social Withdrawal	25.4	24.2	0.696	19.3	18.3	0.649	1.03 (0.91)	0.94 (0.97)	0.85 (0.91)	0.77 (0.89)
Wishful thinking	20.6	24.6	0.238	13.7	14.6	0.746	0.56 (0.87)	0.92 (0.94)	0.62 (0.83)	0.69 (0.79)
Resignation	64.7	51.2	< 0.001	57.7	53.5	0.248	1.89 (1.04)	1.63 (1.03)	1.70 (1.10)	1.62 (1.08)
Sum scores							4.85 (2.12)	4.55 (2.37)	4.10 (2.35)	4.08 (2.36)
Negative										
Self criticism	4.8	8.7	0.054	4.7	6.6	0.280	0.34 (0.66)	0.41 (0.75)	0.25 (0.58)	0.31(0.61)
Blaming others	6.4	5.6	0.670	7.0	6.6	0.838	0.41 (0.69)	0.42 (0.64)	0.38 (0.72)	0.43 (0.72)
Sum scores							0.75 (1.08)	0.83 (1.08)	0.63 (1.03)	0.74 (1.09)

### Associations between stress, coping strategies, and mental health status over time

We further examined whether longitudinal or cross-sectional associations existed between perceived stresses due to the COVID-19 pandemic, coping strategies in 2020 and 2021, and mental health outcomes (anxiety, depression, ODD and ADHD) in 2021. For each outcome, we generated and evaluated two multiple regression models. Table 5 summarizes the results for anxiety and depression symptoms and Table 6 the results for ADHD and ODD. Model 1 used the sum of stresses perceived secondary to COVID-19 and coping strategies assessed in 2020 to predict mental health outcomes in 2021 (longitudinal relationship), while Model 2 used the sum of perceived stresses and coping strategies in 2021 to predict mental health outcomes in 2021. We further adjusted for child gender, parental reports of a poor relationship with their partner during the 1st lockdown, and pre-existing psychiatric problems in the adolescent. We used summation scores for negative, avoidant, and active coping as predictors. Cognitive restructuring exhibited an effect on anxiety and depression symptoms that was directionally opposite to other active coping strategies, improving rather than worsening symptoms.

**Table 5:** Multiple regression model predicting anxiety and depression symptoms in 2021

Predictor	Anxiety symptoms				Depression Symptoms			
	Model 1		Model 2		Model 1		Model 2	
	*β *(SE)	*P*	*β *(SE)	*P*	*β *(SE)	*P*	*β *(SE)	*P*
Intercept	0.147 (0.510)	0.773	− 0.236 (0.427)	0.581	1.374 (0.741)	0.064	− 0.235 (0.626)	0.708
Male	− 0.968 (0.228)	< 0.001	− 0.858 (0.204)	< 0.001	− 1.529 (0.330)	< 0.001	− 1.278 (0.299)	< 0.001
Parents’ poor relationship with partners during pandemic	2.475 (0.538)	< 0.001	2.030 (0.489)	< 0.001	1.219 (0.330)	0.119	0.578 (0.716)	0.420
Previous psychiatric problem	1.754 (0.354)	< 0.001	1.420 (0.315)	< 0.001	2.266 (0.513)	< 0.001	1.751 (0.462)	< 0.001
Sum of stress 2020	0.113 (0.019)	< 0.001			0.075 (0.028)	0.007		
Negative coping 2020	0.326 (0.116)	0.005			0.741 (0.169)	< 0.001		
Avoidant coping 2020	0.091 (0.056)	0.108			0.227(0.082)	0.006		
Cognitive restructuring2020	− 0.279 (0.136)	0.041			− 0.212(0.197)	0.282		
Active coping 2021	0.080 (0.062)	0.200			0.059 (0.090)	0.515		
Sum of stress 2021			0.108 (0.017)	< 0.001			0.132 (0.025)	< 0.001
Negative coping 2021			0.835 (0.100)	< 0.001			0.867 (0.146)	< 0.001
Avoidant coping 2021			0.140 (0.050)	0.004			0.413 (0.072)	< 0.001
Cognitive restructuring 2021			− 0.275 (0.118)	0.020			− 0.550(0.173)	0.002
Active coping 2021			0.079 (0.056)	0.156			0.010 (0.082)	0.899
Adjusted *R*^2^	0.263		0.407		0.197		0.343

**Table 6:** Multiple regression model predicting ADHD and ODD symptoms in 2021

Predictor	ADHD symptoms				ODD symptoms			
	M1		M2		M1		M2	
	*β *(SE)	*P*	*β *(SE)	*P*	*β (*SE)	*P*	*β *(SE)	*P*
Intercept	1.245 (0.309)	0.773	0.799 (0.277)	0.004	0.989 (0.255)	< 0.001	0.705 (0.231)	0.002
Male	0.137 (0.147)	0.349	0.205 (0.142)	0.150	− 0.124 (0.121)	0.306	− 0.059 (0.118)	0.617
Parents’ poor relationship with partners during pandemic	− 0.046 (0.347)	0.895	− 0.162 (0.338)	0.632	0.537 (0.286)	0.060	0.347 (0.282)	0.219
Previous psychiatric problem	1.176 (0.224)	< 0.001	1.086 (0.219)	< 0.001	0.645 (0.185)	< 0.001	0.553 (0.182)	0.003
Sum of stress 2020	0.049 (0.012)	< 0.001			0.037 (0.010)	< 0.001		
Negative coping 2020	0.214 (0.074)	0.004			0.099 (0.061)	0.107		
Avoidant coping 2020	0.073 (0.036)	0.042			− 0.033(0.030)	0.268		
Cognitive restructuring + active coping 2020	− 0.075 (0.033)	0.025			0.029(0.027)	0.283		
Sum of Stress 2021			0.066 (0.012)	< 0.001			0.054 (0.010)	< 0.001
Negative coping 2021			0.158 (0.069)	0.0231			0.109 (0.058)	0.059
Avoidant coping 2021			0.106 (0.034)	0.002			− 0.010 (0.028)	0.718
Cognitive restructuring + active coping 2021			− 0.084 (0.031)	0.008			− 0.021 (0.026)	0.423
Adjusted *R*^2^	0.116		0.167		0.073		0.105	

For anxiety symptoms in 2021, girls, parents’ poor relationship with partners, and the child’s pre-existing psychiatric problems were significantly associated with higher anxiety symptoms. Perceived stress in 2020 and 2021 also was significantly associated with higher symptoms of anxiety. Use of both negative and avoidant coping strategies in either 2020 or 2021 also was significantly associated with increased anxiety symptoms in 2021. On the other hand, using cognitive restructuring in both 2020 and 2021 was associated with lower anxiety symptoms in 2021. No associations between other active coping strategies and anxiety symptoms were significant.

For depression symptoms in 2021, girls and pre-existing psychiatric problems were linked to higher depression symptoms. Perceived stress both in 2020 and 2021 also was associated with higher depression symptoms. Both negative and avoidant coping strategies in 2020 and 2021 were associated with higher depression symptoms. Use of cognitive restructuring in 2020 was not significantly associated with decreased depression symptoms. On the other hand, use of cognitive restructuring in 2021 was associated with less severe depression symptoms in 2021.

Gender and parental relationships were not associated with either ADHD or ODD symptoms in 2021. However, pre-existing psychiatric problems were significantly linked to greater symptoms of both. Perceived stress, both in 2020 and 2021, was significantly associated with higher ADHD and ODD symptoms. Negative and avoidant coping strategies used in both 2020 and 2021 were associated with higher ADHD symptoms, while active positive coping strategies (including cognitive restructuring) were associated with lower ADHD symptoms. None of the coping strategies was significantly linked to ODD symptoms.

## Discussion

This is the first study to examine mental health in both the first and second year of the COVID-19 pandemic approximately 1.5 years after pandemic onset in a large national sample of Swiss adolescents. Several findings are noteworthy.

Previous studies indicated a larger impact of the pandemic on girls [6, 14, 18, 35–37], including large longitudinal studies with pre-pandemic comparison data [41–43]. Although we lack pre-pandemic comparison data, [6, 14, 18, 35–37], our results showed that girls perceived more COVID-related stress and our study additionally shows that this extends to the second pandemic year. Girls’ perceived stress due to troubles getting medical care or mental health services increased from 2020 to 2021 whereas no such increase was found in boys.

The male/female ratios of ADHD and ODD that we found warrant closer inspection as they differ from what is often reported in the pre-pandemic literature. First, the ratios do not seem to be mere methodological artifacts since we used validated instruments and a large random sample that was drawn from a national data base. There is no obvious reason to expect a higher prevalence of ADHD and ODD or a reason why there should be an especially high number of girls with ADHD and ODD in this data base. Study drop-out is also an unlikely explanation since similar prevalence rates and male–female ratios were already found in the analysis of the baseline data [18]. It should also be noted that these are self-reported symptoms from general population and not clinical diagnoses. Second, concerning ADHD, the results are not that unusual. Evidence suggests that ADHD is underdiagnosed in girls and that the male–female-ratio observed in community and general population samples is accordingly smaller than in clinical samples [44, 45]. Furthermore, among young adults aged around 18–19 years, studies repeatedly observed higher ADHD rates among females [46–48]. Third, there is evidence of a detrimental impact of the pandemic on externalizing symptoms [49, 50]. If girls experience more pandemic-related stress, as we observed in our study, the smaller male/female-ratio could result from such higher stress. Indeed, a large longitudinal study that included pre-pandemic comparison data indicated a stronger effect of the pandemic on girls’ total mental health difficulties, emotional symptoms, hyperactivity symptoms, and conduct problems as compared to boys [42]. Another longitudinal study with pre-pandemic data reported similarly evidence that the Covid-19 pandemic had a negative impact on girls’ but not on boys’ externalizing difficulties [41]. Considering the unprecedented and extraordinary nature of the pandemic, more research is needed to clarify the pandemic’s impact on externalizing symptoms, especially in girls.

Girls also more often used the avoidant and maladaptive coping style of ‘wishful thinking’, and they were more likely to stop using the beneficial coping style of ‘cognitive restructuring’ in 2021 (40% in 2021 compared to 52% in 2020, whereas with 49.3% and 51.2% in 2020 and 2021, respectively, no such change was evident among boys).

It is less clear what mechanisms explain the gender difference. The mentioned difference in coping offers a potential starting point. Thorisdottir et al. suggested that hormonal influences during puberty might increase adolescent girls’ sensitivity to interpersonal stressors and that they are more likely to engage in behaviors that specifically exacerbate depressive symptoms, such as extensively discussing problems in dyadic relationships [43]. Other studies found that both women and girls worried more about pandemic-related health concerns, such as, for example, getting and spreading the virus [37, 51], and such worries might well have a detrimental effect on mental health. Similarly, Lelek-Kratiuk and Szczygieł found that women were more likely to evaluate COVID-19-related lockdown as a threat or harm and loss [52], a finding that might well apply to girls as well. Kaltschik et al. offered some hypotheses including girls’ stronger reliance on a social network for emotional support with these networks being reduced by Covid-19-related restrictions of social life, a larger reduction of physical activity levels among girls, and girls’ higher smartphone usage during the pandemic [53]. The hypothesis of a loss of social networks is consistent with a study among UK adults that found that perceived loneliness and loss of social interaction explained a substantial portion of gender differences in pandemic-related mental health [54]. However, a full elucidation of the gender differences is pending. Future research should examine this topic in detail.

Early large-scale screening for adolescents with a pre-existing psychiatric problem—for example, in schools and clinical settings—may be of value. These adolescents had consistently worse mental health in the second pandemic year 2021 than youth without such a history. They scored higher on symptoms of anxiety, depression, ADHD, and ODD, consistent with previous reports which indicate that young people with pre-existing mental health problems may be more affected [6, 12, 15, 16, 22] including the second pandemic year.

COVID-19 related stress during the lockdown period in 2020 longitudinally predicted subsequent symptoms of anxiety, depression, ADHD and ODD in the summer of 2021. This suggests that the stress experienced at the beginning of the pandemic exerted an enduring detrimental effect on our youths’ mental health in 2021, consistent with previous literature indicating the detrimental effect of pandemic-related stress on adolescents’ mental health [4, 8–11, 13, 14, 35, 36]. This could be due, for example, to social isolation and loneliness during lockdowns [55] or to the accumulation of further stress during the ongoing pandemic. In addition, the ongoing pandemic-related stress may delay or even hinder recovery from mental health problems developed earlier in the pandemic.

Coping via cognitive restructuring (e.g., “looking at the positive side of things”) during the lockdown period in 2020 was longitudinally associated with less severe anxiety symptoms, and cross-sectionally in 2021 with less severe depression symptoms, in line with literature documenting this as a fruitful coping behavior [13, 19, 56], with cognitive restructuring being an integral part of psychotherapy for anxiety and depression [57, 58], and with a large international study conducted to evaluate the efficacy of a re-appraisal intervention among adults during the COVID-19 pandemic [59] that found that a simple intervention that encouraged participants to refocus “on whatever good aspects may be found in a situation” reduced negative emotions and increased positive emotions. It is likely that the cognitive restructuring style of coping is particularly important during the COVID-19 pandemic, since an individual’s ability to influence the pandemic itself is limited. That is, despite being able to personally engage in protective behaviors—like social distancing and mask wearing—individuals likely view the pandemic, as a whole, as being well beyond their control. In such a situation, striving for control might not be a productive coping behavior [60], whereas altering one’s appraisal of one’s situation may become relatively powerful. Cognitive restructuring was one of the two coping strategies most often used by our sample, suggesting that the youths we studied were intuitively aware of its relevance within the context of a pandemic. Applying a large-scale intervention to train individuals in this coping behavior—for example, in schools and other educational institutions—might increase the percentage of youths who use the strategy which could, in turn, save some of them from mental health problems and counter some of the increased frequency of stressors that can occur during a pandemic.

Contrary to its effect on anxiety and depression symptoms, cognitive restructuring alone was not predictive of symptoms of either ADHD or ODD. This said, the entire package of active coping strategies—including cognitive restructuring, problem solving, feeling expression, calming down, and social support—was linked to ADHD symptoms, both longitudinally and again cross-sectionally when assessed in 2021. It appears, therefore, that using several coping strategies together permitted individuals to reduce their symptoms of ADHD. This seems to further suggest that, whereas for anxiety and possibly depression, teaching single coping strategies like cognitive restructuring could help adolescents, teaching youths a package of adaptive coping strategies may be necessary for other problems like ADHD.

Engaging in negative coping (self-criticism, blaming others) and avoidant coping was associated with more severe symptoms of anxiety, depression, and ADHD, in line with literature that has documented such coping styles as being associated with mental health problems [56, 61]. Therefore, early large-scale screening for maladaptive coping behaviors may be a fruitful public health initiative. Furthermore, monitoring adolescents for such coping behaviors may allow caregivers, teachers, social workers, clinicians, and whoever else is in regular contact with adolescents to identify those who are at heightened risk. Since such coping behaviors generally fail to overcome whatever stressors exist (as with problem-focused coping and seeking social support) or to re-orient an individual’s perception of their stressful situation (as with re-appraisal and emotion regulation), the stressor will continue to exert its detrimental effects. As a consequence, youths exhibiting predominantly such coping behaviors are at risk of developing mental health problems during a pandemic like COVID-19 and should, therefore, be identified and supported.

Overall, considering the ongoing and continuously elevating detrimental effects of the COVID-19 pandemic suggests that, during future pandemics or similar society-wide crises, early public health interventions might be of considerable value. This includes early, large-scale screening of adolescents at risk of developing mental disorders (e.g., based on pre-pandemic mental health status or maladaptive coping styles) and of adolescents who exhibit subclinical symptomatology. A possibly large number of youth could be reached with scalable interventions such as the cognitive restructuring intervention by Wang et al. [59] or single-session psychiatric interventions for both adolescents [62, 63] and their caregivers [64]. Potentially, such early large-scale interventions could prevent some of the over-burdening of the mental health service system that was observed in the course of the COVID-19 pandemic. Implementing a cycle of screening and early intervening when necessary might offer lasting protection against continuing pandemic stress.

### Strengths and limitations

The present study used a large national sample of adolescents that included respondents from all three language regions in Switzerland, in contrast to many other studies that used convenience or snowball sampling. The longitudinal data allowed examining both short- and long-term effects of COVID-19 related stress and coping. On the other hand, there are several study limitations. First, in 2020 the survey was conducted 1–3 months after the lockdown due to the inevitable delay of obtaining ethical approval and funding. Hence, the results might be subject to recall bias. Second, the survey was conducted online rather than in person, and the instruments measured symptoms of mental health problems and cannot establish a diagnosis. Third, as in any study with informed consent, we cannot rule out some bias due to self-selection. Also, as in any longitudinal study there might be attrition bias. As mentioned in the methods section, girls and possibly those who perceived more COVID-19-related stress were more likely to be non-participants at follow-up, whereas, however, age, language region, nationality, any mental disorder symptom, previous psychiatric problem, parent’s education, and parent’s depression were not related to non-participation. Fourth, adolescents might have completed the questionnaire in the presence of their parents, possibly inducing bias. Fifth, the majority of variables were obtained from the same informants, possibly inflating relationships due to shared method variance. Finally, generalizing our results to other countries should be done with caution due to the different pandemic, political, and economic situations.

## Conclusions

Girls appear to have been more affected by the pandemic than boys and youths with pre-existing psychiatric problems appear to be an especially vulnerable group. COVID-related stress during the lockdown period in 2020 longitudinally predicted subsequent symptoms of anxiety, depression, ADHD and ODD. Healthcare and school professionals should support to identify vulnerable groups and adolescents showing resignation and using negative and avoidant coping strategies and train youths to use more active as well as positive coping strategies.

## Supplementary Information

Below is the link to the electronic supplementary material.

Supplementary file1 (DOCX 28 KB)
