# Supplementary material for: Perceived stress, coping strategies, and mental health status among adolescents during the COVID-19 pandemic in Switzerland: a longitudinal study
Source: Eur Child Adolesc Psychiatry. 2022 Dec 14;32(6):937–49. doi: 10.1007/s00787-022-02119-y (PMC9749639; doi:10.1007/s00787-022-02119-y)
Supplement: Supplementary file 1 — Supplementary file1 (DOCX 28 KB) [file 787_2022_2119_MOESM1_ESM.docx]

**Supplementary material**

Supplement table 1 Logistic regression predicting **non-participation** at follow-up ( N=1146)

|  | Odds ratio (95% CI) | | P-value |
| --- | --- | --- | --- |
| Girls | 1.33 | (1.05,1.68) | **0.020** |
| Age | 1.04 | (0.97,1.12) | 0.301 |
| Language Region |  |  |  |
| French | 1.00 | --- | --- |
| GermAn | 1.14 | (0.88,1.48) | 0.327 |
| Italian | 0.68 | (0.44, 1.05) | 0.079 |
| Nationality(swiss) | 1.04 | (0.65,1.66) | 0.886 |
| Any mental problem-children | 0.98 | (0.76,1.28) | 0.905 |
| Pre-existing mental illness | 1.08 | (0.75,1.57) | 0.682 |
| Sum of perceived stress | 1.02 | (1.00,1.04) | 0.054 |
| Education (parent)-high | 0.89 | (0.70,1.14) | 0.344 |
| depression (parent) | 0.80 | (0.51, 1.26) | 0.337 |

Supplement Table 2 GEE models to test the gender and time effects in perceived COVID-19-related stress

|  | Time(2021) | | girl | | Time*girl | |
| --- | --- | --- | --- | --- | --- | --- |
|  | OR [95%CI] | P | OR [95%CI] | P | OR [95%CI] | P |
| 1. My family has experienced financial problems | 0.76[0.39, 1.48] | 0.428 | 0.78[0.37, 1.66] | 0.524 | 2.38[0.95, 5.94] | 0.063 |
| 1. Unable to spend time in person with my friends or family | 0.69[0.51, 0.93] | 0.015 | 1.42[1.00, 2.02] | 0.052 | 1.32[0.85, 2.03] | 0.217 |
| 1. Unable to participate in social activities and normal routines | 0.74[0.56, 0.99] | 0.043 | 1.35[0.96, 1.91] | 0.088 | 1.14[0.74, 1.76] | 0.538 |
| 1. Having to change, postpone, or cancel important plans or events | 0.84[0.63, 1.18] | 0.243 | 1.85[1.31, 2.62] | <0.001 | 1.08[0.70, 1.67] | 0.732 |
| 1. Challenges at home or with others | 1.20[0.65, 2.23] | 0.564 | 1.96[1.02, 3.76] | 0.043 | 1.34[0.64, 2.83] | 0.437 |
| 1. My family has experienced trouble getting groceries or other needed supplies | 0.55[0.19, 1.54] | 0.252 | 1.90[0.81, 4.47] | 0.140 | 1.15[0.31, 4.25] | 0.831 |
| 1. Watching or hearing distressing news reports about COVID-19 | 0.52[0.33, 0.81] | 0.004 | 1.75[1.15 ,2.66] | 0.009 | 1.40[0.80, 2.46] | 0.242 |
| 1. Not being sure about myself or someone close to me getting COVID-19 | 0.80[0.56, 1.16] | 0.241 | 1.62[1.08, 2.44] | 0.020 | 0.89[0.53, 1.48] | 0.653 |
| 1. Myself or someone close to me having symptoms or being diagnosed with COVID-19 | 0.58[0.40, 0.85] | 0.005 | 1.39[0.93, 2.07] | 0.104 | 1.35[0.80, 2.28] | 0.262 |
| 1. Trouble getting medical care or mental health services | 0.71[0.36, 1.39] | 0.314 | 1.57[0.83, 2.99] | 0.166 | 2.35[1.04, 5.29] | 0.039 |
| 1. Not being sure about when COVID-19 will end or what will happen in the future | 0.96[0.69, 1.33] | 0.806 | 2.07[1.41, 3.03] | <0.001 | 0.79[0.50, 1.25] | 0.313 |
| 1. Difficulty completing my school/ work responsibilities online | 0.80[0.55, 1.15] | 0.225 | 0.96[0.62, 1.49] | 0.852 | 0.86[0.49, 1.51] | 0.597 |
| 1. Unable to complete educational or work requirements | 0.81[0.53, 1.23] | 0.317 | 1.09[0.68, 1.76] | 0.721 | 1.24[0.68, 2.26] | 0.485 |
| 1. Needing to take on greater family and /or work responsibilities | 0.75[0.41, 1.35] | 0.331 | 1.21[0.64, 2.27] | 0.557 | 1.41[0.63, 3.17] | 0.406 |
| Sum of stress scores | -1.44[-2.15, -0.72] | <0.001 | 2.20[1.09, 3.30] | <0.001 | 0.36[-0.75, 1.46] | 0.529 |

Supplement table 3 Association between COVID-19-related stressors in 2021 and mental health 2021

|  | **Mental health 2021** | | | | |
| --- | --- | --- | --- | --- | --- |
| **COVID-19-related stressors** | **Depression** | **Anxiety** | **ADHD** | **ODD** | **Any** |
| **Girls** | **OR**  **(95% CI)** | **OR**  **(95% CI)** | **OR**  **(95% CI)** | **OR**  **(95% CI)** | **OR**  **(95% CI)** |
| **My family has experienced financial problems** | 8.38*  (2.89, 24.35) | 2.46  (0.83, 7.28) | 3.13*  (1.26, 7.78) | 2.39  (0.91, 6.29) | 2.29  ( 0.93, 5.66) |
| **Challenges at home or with others** | 20.22*  (7.11, 57.53) | 6.76*  (2.96, 15.42) | 5.21*  (2.53, 10.76) | 5.74*  (2.72, 12.12) | 5.88*  ( 2.71, 12.80) |
| **Trouble getting medical care or mental health services** | 4.86*  (1.83, 12.91) | 4.31*  (1.85, 10.04) | 2.57*  (1.23, 5.37) | 2.59*  (1.18, 5.65) | 2.59*  (1.18, 5.65) |
| **Boys** |  |  |  |  |  |
| **My family has experienced financial problems** | 1.62  (0.20, 13.36) | 2.98  (0.88, 10.02) | 2.24  (0.72, 6.95) | 4.94*  (1.55, 15.75) | 4.58*  (1.49, 14.07) |
| **Challenges at home or with others** | 10.83*  (3.20, 36.62) | 10.08*  (3.78, 26.90) | 3.89*  (1.51, 10.04) | 4.19*  (1.48, 11.88) | 7.59*  (2.64, 21.78) |
| **Trouble getting medical care or mental health services** | 1.76  (0.21, 14.61) | 3.32  (0.97, 11.37) | 3.55*  (1.15, 10.98) | 1.45  (0.31, 6.86) | 2.88  (0.94, 8.83) |

*P<0.05; if the 95% CI does not contain the value 1, the p-value is less than 0.05.
